# Augmented photocatalysis induced by 1T-MoS_2_ bridged 2D/2D MgIn_2_S_4_@1T/2H-MoS_2_ Z-scheme heterojunction: mechanistic insights into H_2_O_2_ and H_2_ evolution†

**DOI:** 10.1039/d3na00912b

**Published:** 2024-01-15

**Authors:** Sarmistha Das, Lopamudra Acharya, Lijarani Biswal, Kulamani Parida

**Affiliations:** a Centre for Nano Science and Nano Technology, Institute of Technical Education and Research, Siksha ‘O’ Anusandhan University Bhubaneswar-751030 India kulamaniparida@soa.ac.in paridakulamani@yahoo.com +91-674-2350642 +91-674-2351777

## Abstract

In the realm of composite photocatalysts, the fusion of the co-catalyst effect with interfacial engineering is recognized as a potent strategy for facilitating the segregation and migration of photo-induced charge carriers. Herein, an innovative mediator-based Z-scheme hybrid, *i.e.* MIS@1T/2H-MoS_2_, has been well designed by pairing MIS with 1T/2H-MoS_2_*via* a facile hydrothermal strategy as a competent photocatalyst for H_2_O_2_ and H_2_ generation. The co-catalyst, *i.e.* metallic 1T-phase bridging between semiconducting 2H-MoS_2_ and MIS, serves as a solid state electron mediator in the heterostructure. Morphological findings revealed the growth of 1T/2H-MoS_2_ nanoflowers over MIS microflowers, verifying the close interaction between MIS and 1T/2H-MoS_2_. By virtue of accelerated e^−^/h^+^ pair separation and migration efficiency along with a proliferated density of active sites, the MMoS_2_-30 photocatalyst yields an optimum H_2_O_2_ of 35 μmol h^−1^ and H_2_ of 370 μmol h^−1^ (ACE of 5.9%), which is 3 and 2.7 fold higher than pristine MIS. This obvious enhancement can be attributed to photoluminescence and electrochemical aspects that substantiate the diminished charge transfer resistance along with improved charge carrier separation, representing a good example of a noble metal-free photocatalyst. The proposed Z-scheme charge transfer mechanism is aided by time-resolved photoluminescence (TRPL), XPS, radical trapping experiments, and EPR analysis. Overall, this endeavour provides advanced insights into the architecture of noble metal-free Z-scheme heterostructures, offering promising prospects in photocatalytic applications.

## Abstract 2

This work involves the fabrication of a 1T phase mediated MIS@1T/2H-MoS_2_ Z-scheme hybrid for accelerated photocatalytic H_2_O_2_ and H_2_ production.

## Graphical abstract

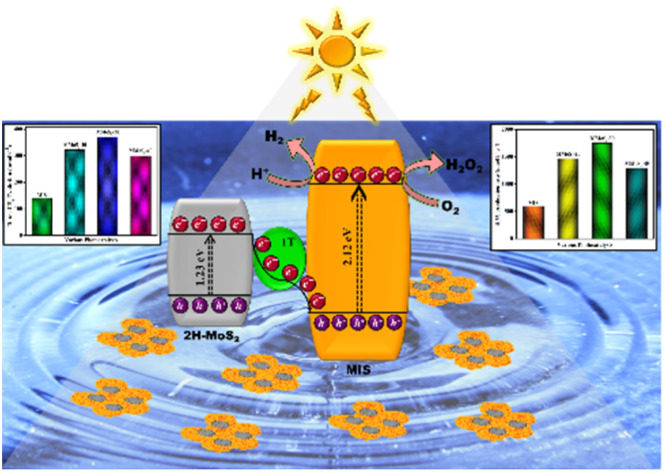

## Introduction

The significant expansion of industrialization in the current world is giving rise to the two most pressing global issues: energy crisis and environmental pollution.^1,2^ Consequently, research communities are actively seeking a long term and unchanging resolution to achieve an eco-friendly society devoid of pollution, all while reducing our reliance on non-renewable energy resources.^3^ Hydrogen peroxide (H_2_O_2_), recognized as an environmental friendly oxidant, has garnered significant interest among the leading researchers owing to its use in various domains, including bleaching, medical sterilization, food and paper making, waste water disinfection and chemical processing, with approximately 2.2 million tons of annual demand.^4,5^ H_2_O_2_ has also attracted considerable attention as a novel energy carrier for fuel cells as it is water soluble and favourable to be implemented in a single-compartment cell for generating electricity.^6,7^ Several techniques involving alcohol oxidation, anthraquinone process, electrochemical synthesis, direct fabrication from hydrogen and oxygen gas mixtures have been developed for H_2_O_2_ production.^8–11^ Nonetheless, certain drawbacks associated with the above outlined synthesis methods prompts the need for substantial energy input, elevated hazard of explosion from combination of H_2_/O_2_ gases, and extensive solvent use.^12^ At present, the industrial production of H_2_O_2_ relies on the anthraquinone method, consisting of two successive high-energy oxidation and hydrogenation steps considering catalysts based on noble metals, which is both an energy intensive and non-eco-friendly process.^8,13^ Therefore, the need of embracing a green and ecologically sustainable approach is highly on demand.^14–16^ Due to remarkable attributes of minimal energy expenditure, enhanced safety measures, and negligible pollution, production of H_2_O_2_ from oxygen and water using photocatalysts *via* artificial photosynthetic process has currently been perceived.^17–19^ Furthermore, hydrogen (H_2_) holding is promising as a viable eco-friendly alternative to the diminishing non-renewable energy sources and serves as a green, clean and sustainable energy source.^20–22^ Researchers are drawn to the intriguing potential of photocatalytic H_2_ generation using semiconductors *via* water splitting owing to its simple process and economic feasibility.^23–25^ Several semiconductor oriented materials have been optimized for enhanced photocatalytic generation of H_2_O_2_ as well as H_2_.

In this regard, attention has been directed towards 2D-transition metal dichalcogenides (TMD) and ternary metal chalcogenides such as thiospinel-type materials. Recently, 2D molybdenum disulphide (MoS_2_), *i.e.*, a member of the TMD family, has emerged as a cost-effective and readily available substitute to Pt-based materials owing to its abundant active sites, excellent optoelectronic and structural features along with narrow band gap.^26,27^ MoS_2_ exhibits a distinct tri-layered structure in a unique S–Mo–S pattern, where one Mo layer is stacked between two S layers, featuring unsaturated S-atoms at its exposed edges as reactive sites.^26,28^ Typically, two primary phases of MoS_2_ are explored in depth: the octahedral metastable metallic 1T-phase and the stable trigonal prismatic semiconducting 2H-phase. Regardless of their respective interesting benefits, phase engineering has lately been focussed on where the 1T-phase is incorporated into the 2H-phase (*i.e.*, 1T/2H-MoS_2_) for high-end photocatalytic results.^28,29^ Integrating 1T-MoS_2_ into the 2H-phase exponentially augments electronic conductivity by 10^7^ times, thus significantly bolstering charge transfer dynamics and reducing electrical transport loss. The developed 1T/2H-MoS_2_ nanostructure boasts a high density of active sites on both the basal planes and edges suitable for effective photocatalytic performance.^30,31^ The semiconducting 2H-MoS_2_ facilitates photon adsorption and the 1T-MoS_2_ functions as an e^−^ reservoir. Moreover, the construction of 2D–2D semiconductors having noble metal-free co-catalyst profoundly enhances the photocatalytic behaviour due to stronger interfacial interaction between two semiconductors, good catalytic dispersion, and effective segregation of photo-generated exciton pairs.^32,33^ Hence, the establishment of a system with a semiconductor having a band structure compatible with 1T/2H-MoS_2_ is demanding as it enhances light absorption and charge carrier transfer efficiency.

Magnesium indium sulphide (MgIn_2_S_4_), a ternary metal sulphide resembling that of spinel, features Mg and In at the core of tetrahedron and octahedron, respectively. MgIn_2_S_4_, owing to its exceptional features, including narrow band gap, good optoelectronic property, and remarkable chemical stability, has achieved wide utility in fields like photoreduction of heavy metal ions, water splitting (H_2_ evolution), environmental pollutant removal, and more.^34,35^ However, in comparison to other efficient photocatalysts, the photocatalytic efficacy of ternary metal chalcogenide is somewhat constrained on account of photo-corrosion, weak e^−^/h^+^ pair separation efficiency, and poor cycle stability test.^34,36^ To address these flaws, designing hybrid semiconductors has emerged as a productive approach. Moreover, from a pragmatic perspective, an integrated heterostructure, including two discrete semiconductors closely interlocked through charge transfer, is deemed more favourable. In contrast to the classic type-II heterojunctions, bio-inspired Z-scheme heterojunctions not only enhance light harvesting features and the spatial dispersion of charge carriers but also maintain the elevated redox potentials of photo-induced h^+^ and e^−^, which is crucial for H_2_O_2_ generation.^32,37^ In this context, 1T/2H-MoS_2_@BCN synthesized *via* hydrothermal strategy was applied towards TCH degradation and H_2_ evolution (290 μmol h^−1^) in our previously reported work. Although it gives fascinating results, MgIn_2_S_4_@1T/2H-MoS_2_ is designed to further promote the photocatalytic activity of 1T/2H-MoS_2_.

In light of the above view, using an *in situ* hydrothermal method, we have made an attempt to design a series of mediator-based Z-scheme photocatalysts (*i.e.*, MMoS_2_-*x*, *x* = wt% of 1T/2H-MoS_2_) by combining 1T/2H-MoS_2_ nanoflowers with MgIn_2_S_4_ microflowers. The coupling effect among individual materials is verified by subjecting the system to visible light irradiation and assessing its performance towards photocatalytic H_2_O_2_ and H_2_ production. Effective segregation of e^−^/h^+^ pairs, close interfacial interaction, and a broader range of visible light absorption influence the improved photocatalytic behaviour of this novel heterostructure. Also, the involvement of 1T-phase, a cost-effective and abundantly found noble metal-free co-catalyst, reduces the recombination efficiency of the exciton pairs. The growth of 1T/2H-MoS_2_ nanoflowers over MgIn_2_S_4_ microflowers results in elevated density of active sites, leading to impressive photocatalytic activity. The current study provides clear guidance regarding the construction and mechanism of the MMoS_2_-30 composite towards accelerated photocatalytic activity *via* the Z-scheme charge transfer route.

## Experimental section

### Materials used

All the chemicals utilised in synthesizing 1T/2H-MoS_2_, MgIn_2_S_4_, and MMoS_2_-*x* composites including ammonium bicarbonate (NH_4_HCO_3_), ammonium molybdate ((NH_4_)_6_Mo_7_O_24_·H_2_O), magnesium nitrate (Mg(NO_3_)_2_·6H_2_O), indium nitrate (In(NO_3_)_3_·*x*H_2_O), and thioacetamide (CH_3_CSNH_2_) were procured from Merck, India. They were all of high purity and analytical grade, requiring no further treatment. Throughout the preparation procedure, ethanol and distilled water were employed as solvents.

### Preparation of MIS microflower

Pristine MIS was prepared *via* a facile hydrothermal strategy. Typically, calculated amounts of Mg(NO_3_)_2_·6H_2_O and In(NO_3_)_3_·*x*H_2_O, as Mg and In precursors, were ultrasonically dispersed in 50 mL distilled water for about 30 min at room temperature. Subsequently, CH_3_CSNH_2_ was gradually added to the aforementioned solution in a ratio of 1 : 2 : 8 (Mg : In : S) and was ultra-sonicated for 30 min, followed by continuous stirring at room temperature for an additional 30 min. Thereafter, the homogenous suspension was transferred into a Teflon-lined vessel and was hydrothermally treated at 180 °C for 12 h. The material underwent centrifugation and was rinsed multiple times with ethanol and distilled water, followed by drying at 60 °C in an oven to obtain the product, *i.e.*, Magnesium Indium Sulphide (denoted as MIS).

### Preparation of MIS@1T/2H-MoS_2_ composites (MMoS_2_-*x*)

**Scheme 1:**
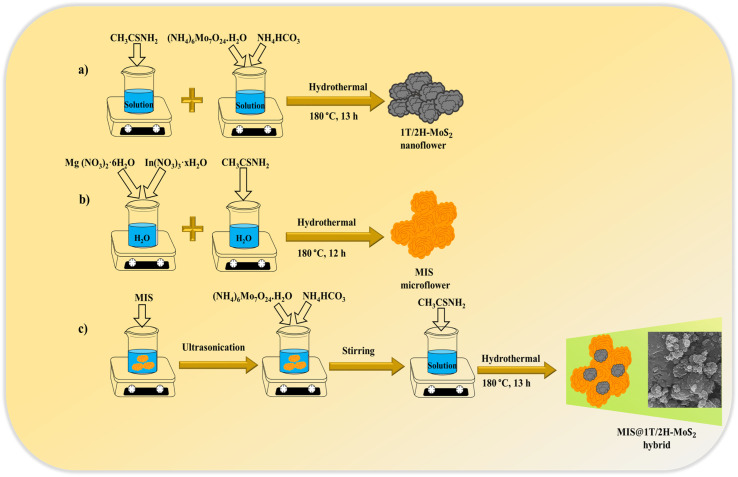
Synthetic routes for (a) 1T/2H-MoS_2_, (b) MIS and (c) MMoS_2_-*x* composites *via* hydrothermal method.

Initially, the synthesized neat MIS (0.3 g) was well-dispersed in 40 mL of distilled water through ultra-sonication for about 1 h, as depicted in Scheme 1. Then, measured quantities of (NH_4_)_6_Mo_7_O_24_·H_2_O and NH_4_HCO_3_ were added to the above solution, which underwent 30 min of stirring. Following this, CH_3_CSNH_2_ was added to the mixture and stirred for 30 more min at room temperature. The resulting suspension was then transferred into a Teflon-lined vessel for a 13 h hydrothermal treatment at 180 °C. Further, the material was collected by centrifugation followed by several washes with ethanol and distilled water. The final sample obtained after drying at 60 °C in vacuum forms the MIS@1T/2H-MoS_2_ composites (referred to as MMoS_2_-*x*), where *x* signifies the wt% of 1T/2H-MoS_2_ (*i.e.*, 20, 30, 40) with respect to MIS. Likewise, by varying the wt% of 1T/2H-MoS_2_ precursors, a set of MIS@1T/2H-MoS_2_ photocatalysts were prepared and designated as MMoS_2_-20, MMoS_2_-30 and MMoS_2_-40. Neat 1T/2H-MoS_2_ was synthesized by adopting the above identical process without adding MIS.

## Results and discussion

### Structural and morphological analysis

**Fig. 1:**
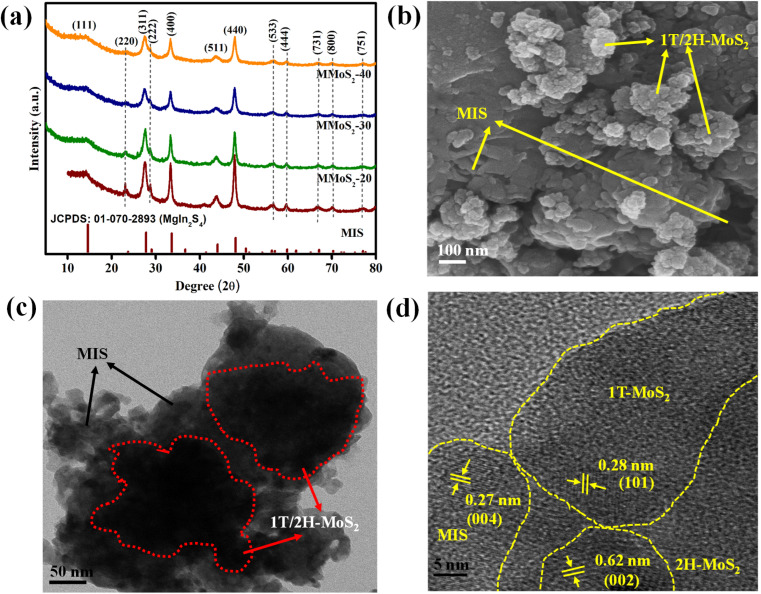
(a) XRD spectra of neat MIS and prepared MMoS_2_-*x* photocatalysts. (b) FESEM image and (c and d) HRTEM images of the MMoS_2_-30 composite.

The XRD analysis of the prepared MIS, MMoS_2_-*x*, and 1T/2H-MoS_2_ nanomaterials shown in Fig. 1a and S1a,† respectively, are performed to interpret their phase purity and crystallographic properties. Typically, in Fig. S1a,† the two distinct diffraction peaks at 9.2° and 18.4° for (002) and (004) lattice planes, respectively, indicate the presence of the 1T phase in 1T/2H-MoS_2_. The other diffraction peaks at 32.3°, 43.0°, and 57.2° can be attributed to the (100), (103), and (110) planes of 1T/2H-MoS_2_, respectively.^30,38^ Concretely, the diffraction patterns for pristine MIS in Fig. 1a display peaks at 14.3°, 23.4°, 27.5°, 28.7°, 33.4°, 43.8°, 48.0°, 56.4°, 59.7°, 67.0°, 70.2° and 77.0° assigned to crystalline planes of (111), (220), (311), (222), (400), (511), (440), (533), (444), (731), (800) and (751), respectively, in reference with JCPDS: 01-070-2893.^35^ The XRD patterns of MMoS_2_-*x* heterojunctions in Fig. 1a closely resembled those of pristine MIS suggesting no significant changes on the crystal planes of MIS by varying the amount of 1T/2H-MoS_2_. Furthermore, as the loading quantity of 1T/2H-MoS_2_ increases, the peak intensity of MIS in MMoS_2_-*x* composites gradually decreases and broadens.^39,40^ Interestingly, no discernible peak is observed for 1T/2H-MoS_2_, indicating excellent homogeneity as well as favourable interaction between the two neat components. The fabricated photocatalysts exhibited a high level of phase purity since no additional peaks of metal oxides, unreacted reactants, and binary sulphides were observed. The Raman spectra of neat MIS, 1T/2H-MoS_2_, and MMoS_2_-30 are presented in Fig. S1b† for further confirmation of the heterostructure formation. According to the previously reported paper,^30^ in neat 1T/2H-MoS_2,_ two peaks at 281.5 and 376.7 cm^−1^ correspond to E_1g_ and E^1^_2g_ of 2H-MoS_2_, whereas peaks positioned at 148.6, 194.5, 212.6, 236.4, and 335.7 cm^−1^ are assigned to the phonon modes of 1T-MoS_2_. Moreover, for pristine MIS, the peaks obtained at 200–400 cm^−1^ are attributed to the vibrational modes of In–S (A_1g_) and Mg–S (E_g_). Owing to the small quantity of 1T/2H-MoS_2,_ the Raman spectra of MMoS_2_-30 are quite identical to that of neat MIS. The peak intensity of MIS in the MMoS_2_-30 composite is reduced by adding 1T/2H-MoS_2_, revealing the formation of a heterostructure.

Field emission scanning electron microscopy (FESEM) and high-resolution transmission electron microscopy (HRTEM) analyses provide information about the morphological aspects of the synthesized materials, *i.e.*, 1T/2H-MoS_2,_ MIS, and their composite MMoS_2_-30. Fig. S1b† displays the FESEM image of pristine MIS showcasing a microflower-like morphology.^35,41^ The FESEM image of 1T/2H-MoS_2_ reveals the formation of nanoflowers resulting from the entwining of individual edges exposing curved nanopetals as observed from previously reported papers.^32,42^Fig. 1b depicts the FESEM image of MMoS_2_-30 illustrating MIS microflowers surrounded by the arbitrary growth of 1T/2H-MoS_2_ nanoflowers that coincides with the HRTEM image in Fig. 1c. As shown in the figure, the MIS microflowers offer a close contact interface with 1T/2H-MoS_2_ nanoflowers forming MMoS_2_-30 hybrid. This intensifies the concentration of active sites, contributing towards enhanced photocatalytic activity. In Fig. 1d, two distinct lattice fringes are evident at 0.28 and 0.62 nm, corresponding to the (101) plane of the 1T phase and (002) plane of the 2H phase, respectively, along with a lattice fringe of 0.27 nm for the (004) plane of MIS.^26,31^ This indicates the co-existence of neat moieties in the MMoS_2_-30 composite. Moreover, the energy dispersive X-ray (EDX) spectra for MMoS_2_-30 presented in Fig. S2a† validate the absence of impurities with the existence of all elements (Mo, S, Mg, and In). The consistent distribution of the constituent elements throughout the MMoS_2_-30 hybrid is presented in Fig. S2(b–e),† supplementing additional support for the strong interaction between the pristine materials.

### XPS analysis

**Fig. 2:**
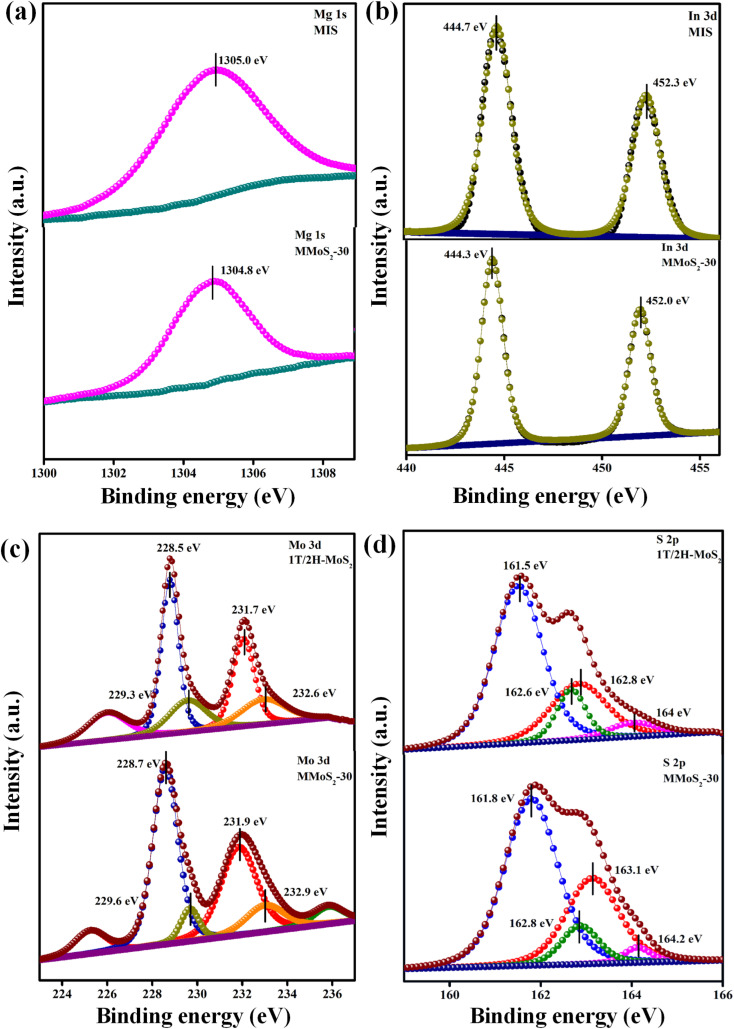
XPS spectra of neat MIS, 1T/2H-MoS_2_ and MMoS_2_-30 composite: (a) Mg 1s, (b) In 3d, (c) Mo 3d, and (d) S 2p.

X-ray photoelectron spectroscopy (XPS), a surface-specific investigative technique, was employed to assess the existence and valence states of various elements within the prepared photocatalysts. The XPS survey spectra for the MMoS_2_-30 composite in Fig. S3† unequivocally evidence the presence of elements from each parent material, including Mg, In, Mo, and S, which is further substantiated by the findings from EDX and elemental mapping. The Mg 1s core level spectrum in neat MIS showed a single broad peak at 1305.0 eV due to the +2 oxidation state (Fig. 2a). As shown in Fig. 2b, the In 3d XPS spectrum is deconvoluted into doublet peaks around 444.7 and 452.3 eV, corresponding to 3d_5/2_ and 3d_3/2_, respectively, of pristine MIS with a peak splitting of 7.68 eV indicating the presence of +3 oxidation state.^35,43^ Moreover, in neat 1T/2H-MoS_2_, the deconvoluted Mo 3d peaks for the 1T phase were obtained at 228.5 and 231.7 eV as well as for the 2H phase at 229.3 and 232.6 eV, corresponding to Mo 3d_5/2_ and 3d_3/2_, respectively (Fig. 2c). According to Fig. 2d, four deconvoluted S 2p peaks of neat 1T/2H-MoS_2_ were positioned at 161.5 and 162.8 eV for the 1T phase along with binding energies of 162.6 and 164.0 eV for the 2H phase, corresponding to S 2p_3/2_ and 2p_1/2_ spin states, respectively.^32,41,44^ The XPS analysis also yields valuable insights into the electron transfer from 1T/2H-MoS_2_ to MIS semiconductor upon forming a solid state Z-scheme heterostructure. This results in slight positive and negative shifts in the binding energy values of 1T/2H-MoS_2_ and MIS in the MMoS_2_-30 composite in contrast to their respective neat counterparts. In comparison to bare MIS, the Mg 1s XPS spectrum in MMoS_2_-30 (Fig. 2a) is shifted to negative binding energy at 1304.8 eV. Concurrently, the two peaks of In 3d spectra in Fig. 2b are negatively shifted to lower binding energy at 444.3 and 452.0 eV compared to neat MIS. Nevertheless, a positive shift is displayed for the Mo 3d spectrum of MMoS_2_-30 in Fig. 2c, with peaks at 228.7 and 231.9 eV regarding the 1T phase as well as at 229.6 and 232.9 eV for the 2H phase, referring to 3d_5/2_ and 3d_3/2_, respectively, than bare 1T/2H-MoS_2_. Simultaneously, S 2p spectra of the MMoS_2_-30 composite also possess a positive shift in binding energies as compared to neat 1T/2H-MoS_2_ (Fig. 2d). The 1T phase has peak values situated at 161.8 and 163.1 eV along with 162.8 and 164.2 eV for the 2H phase designated to the respective spin states of S (*i.e.*, S 2p_3/2_ and S 2p_1/2_). The findings indicated that the electron density of respective materials in the MMoS_2_-30 heterostructure was altered by virtue of electron transfer from 1T/2H-MoS_2_ to MIS *via* 1T-MoS_2_ mediator. Overall, the transfer of electrons from 1T/2H-MoS_2_ to MIS elevates the density of electrons on the MIS surface, thereby shifting the binding energies to lower values (*i.e.*, red shift). Conversely, in 1T/2H-MoS_2_, the binding energy is shifted to higher values (*i.e.*, blue shift) owing to the reduced electron density on the 1T/2H-MoS_2_ surface. Such interaction resulted in an inherent electric field within the system that serves as a driving force enabling the transit of photo-induced e^−^/h^+^ pairs on exposure to visible light.

### Optical properties

**Fig. 3:**
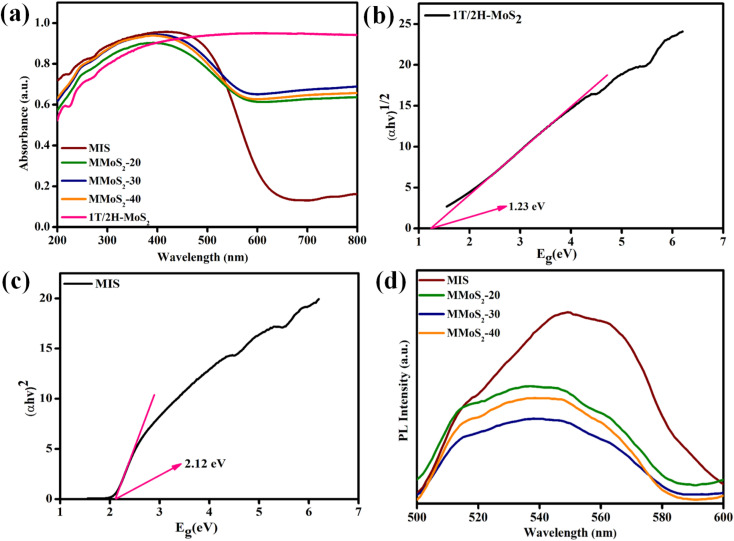
(a) UV-vis DRS of prepared materials. Band gap energy plots of (b) 1T/2H-MoS_2_, (c) MIS and (d) PL spectra of MIS and MMoS_2_-*x* composites.

Considering the significance of light absorption ability in photocatalysis, the optical responses of the prepared neat and composite materials were assessed using UV-vis DRS spectral analysis. According to Fig. 3a, pristine MIS exhibits a distinct absorption threshold in the visible light zone around 635 nm, whereas neat 1T/2H-MoS_2_ displays broad absorption spectra from UV to NIR region.^32,45^ The gradual rise in the loading content of 1T/2H-MoS_2_ in MIS boosts the light-harvesting skill of MMoS_2_-*x* heterostructures accompanied by a red shift towards the visible light region in contrast to bare MIS. This evidences a broader visible light response of the MMoS_2_-*x* hybrid, revealing a firmly established design between two pristine frameworks, which results in an escalated rate of photo-excitation for enhanced photocatalytic behaviour. Moreover, by using the Tauc plot, the band gap energies (*E*_g_) of neat 1T/2H-MoS_2_ and MIS are determined from the Schuster Kubelka–Munk eqn (1) and (2), respectively.

1 *αhν* = *A*(*hν* − *E*_g_)^*n*/2^

2 *αhν* = *A*(*hν* − *E*_g_)^*n*^

The Planck's constant and the absorption coefficient are referred to as *h* and *α*, respectively, with *A* serving as a constant and *n* signifying the electronic transition. *E*_g_ and *ν* represent band gap energy and frequency of light, respectively. With the use of eqn (1) and (2), the calculated band gap energies (*E*_g_) of 1T/2H-MoS_2_ and MIS are 1.23 and 2.12 eV, respectively, as depicted in Fig. 3b and c.^30,46^

Furthermore, the lifetime of photo-induced e^−^/h^+^ pairs is a pre-requisite for improved photocatalytic performance. In this regard, the photoluminescence analysis of the synthesized photocatalysts is represented in Fig. 3d, which provides valuable insights regarding their intricate properties, such as the segregation and recombination efficiency of photo-generated charge carriers. In pristine MIS, an emission peak around 550 nm is observed in contrast to other MMoS_2_-*x* composites. Furthermore, the inclusion of 1T/2H-MoS_2_ into MIS resulted in a significant decrease in the emission peak. With the progressive rise in 1T/2H-MoS_2_ loading, the PL peak intensity of MMoS_2_-*x* photocatalysts shrinks. Among all, the MMoS_2_-30 composite displayed a reduced PL peak intensity, highlighting enhanced charge carrier segregation and migration with a lower rate of recombination.^35^ This can be attributed to the effective coupling between both the neat materials (*i.e.*, 1T/2H-MoS_2_ and MIS), resulting in augmented photocatalytic performance. Additionally, time-resolved photoluminescence (TRPL) analysis is conducted for MIS and MMoS_2_-30 photocatalyst to study the average lifespan of the excitons. The decay curves were fitted using a bi-exponential function as given in eqn (3).

3 Fit = *A* + *α*_1_ exp{−*t*/*τ*_1_} + *α*_2_ exp{−*t*/*τ*_2_}

*α*_1_ and *α*_2_ refer to relative contributions, *τ*_1_ and *τ*_2_ denote the decay lifetimes of the respective materials, and *A* is a constant. The average lifespan (*τ*_avg_) of neat MIS and MMoS_2_-30 composite is computed using eqn (4).4
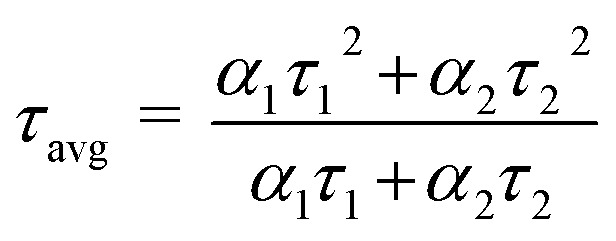


The MMoS_2_-30 hybrid exhibited a shorter average lifetime of 0.61 ns in comparison to neat MIS with an average lifetime of 0.86 ns, as depicted in Fig. S4.† This variation in *τ*_avg_ can be ascribed to the Z-scheme charge dynamics of the heterostructure.^24,32,47^

### Electrochemical properties

**Fig. 4:**
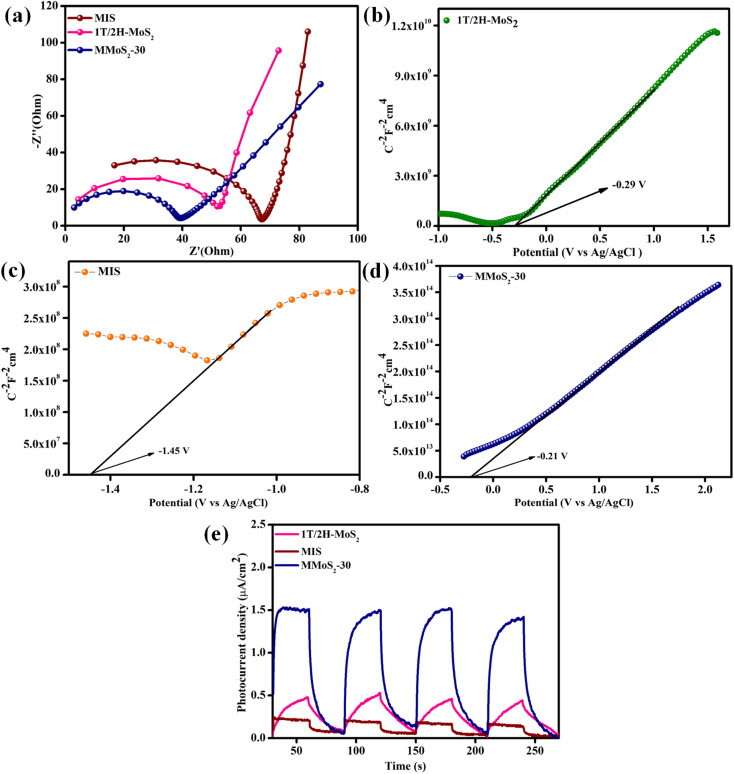
(a) EIS plot of neat components and the prepared MMoS_2_-30 composite. Mott–Schottky plots of (b) 1T/2H-MoS_2_, (c) MIS and (d) MMoS_2_-30. (e) Transient photocurrent measurement of neat 1T/2H-MoS_2_, MIS, and MMoS_2_-30.

The EIS analysis is performed to further explore the effective separation efficiency as well as the rapid transfer of exciton pairs (e^−^/h^+^) in the prepared photocatalysts. The Nyquist plot shows a high-frequency region represented by a semicircle and a low-frequency region denoted by a straight line. Fig. 4a displays the Nyquist plots for 1T/2H-MoS_2_, MIS, and MMoS_2_-30 photocatalysts. As shown in Fig. 4a, the MMoS_2_-30 photocatalyst exhibits a relatively smaller semicircular arc with respect to its parent materials, which implies a declined charge transfer resistance, ultimately leading to improved electrical conductivity of the composite. Also, the EIS observation of the MMoS_2_-30 heterostructure strongly supports the photoluminescence (PL) outcome. Hence, the MMoS_2_-30 composite with respect to its parent materials exhibited higher separation and lower rate of e^−^/h^+^ pair recombination owing to the mediator-based Z-scheme charge transfer dynamics upgrading the photocatalytic activity.^48,49^

Using the Mott–Schottky technique, the nature and flat band potential (*E*_fb_) of the pristine materials can be determined, which is further considered in evaluating the band edge potentials of the semiconducting materials. The MS-plot for both the parent semiconductors, *i.e.*, 1T/2H-MoS_2_, MIS, and MMoS_2_-30 composite in Fig. 4b–d, shows positive slopes with flat band potentials *E*_fb_ such as −0.29, −1.45, and −0.21 V, respectively, revealing themselves to be n-type semiconductors. Subsequently, the conduction band edge potential (*E*_CB_) of both 1T/2H-MoS_2_ and MIS can be evaluated using the following eqn (5),

5 *E*_NHE_ = *E*_Ag/AgCl_ + *E*_Ag/AgCl_ − 0.0591 (7 – pH of the electrolyte)

Using the above equation, the *E*_CB_ of 1T/2H-MoS_2_ and MIS was calculated to be −0.4 and −1.26 V, respectively. Furthermore, considering the obtained *E*_CB_ and *E*_g_, the respective valence band edge potentials (*E*_VB_) for 1T/2H-MoS_2_ and MIS were determined to be 0.7 and 0.86 V by implementing eqn (6),

6 *E*_VB_ = *E*_g_ + *E*_CB_

Moreover, for better insights into the exciton electron–hole separation efficacy, the transient photocurrent analysis is executed for 1T/2H-MoS_2_, MIS, and MMoS_2_-30 under a series of alternative dark and visible light irradiating conditions, as shown in Fig. 4e. Under visible light illumination, the materials exhibited a significant rise in photocurrent that remained relatively stable during the light exposure. However, once the light is turned off, a sudden drop in the current density is observed.^45,50^ According to Fig. 4e, the photocurrent densities achieved for neat 1T/2H-MoS_2_ and MIS were 0.45 and 0.21 μA cm^−2^, respectively, suggesting the poor charge carrier segregation ability of neat materials. However, due to the mediator-based Z-scheme mechanism, the MMoS_2_-30 composite showed a hike in current density around 1.8 μA cm^−2^, 4 and 8.5 times higher than that of 1T/2H-MoS_2_ and MIS, respectively. This affirms the enhanced e^−^/h^+^ pair separation and migration ability in the MMoS_2_-30 heterostructure in contrast to its neat materials.

### Photocatalytic applications

The photocatalytic properties of the as-prepared materials were performed *via* photocatalytic H_2_ generation and H_2_O_2_ production, and the results are precisely discussed as follows.

### Photocatalytic H_2_ evolution

**Fig. 5:**
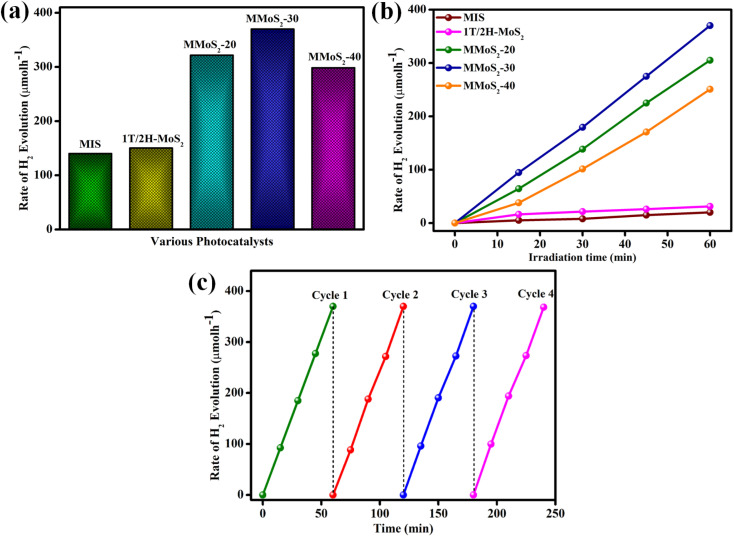
(a and b) Hydrogen production rate of MIS, 1T/2H-MoS_2_, and synthesized MMoS_2_-*x* photocatalysts and (c) reusability experiment of the MMoS_2_-30 composite.

The hydrogen generation rates of the synthesized photocatalysts are displayed in Fig. 5a. The blank experiment evidenced the pivotal role of photocatalysts as well as light during the photocatalytic hydrogen evolution reaction since the absence of both light and photocatalyst shows no hydrogen gas production.^51–53^ A poor hydrogen evolution rate of 140 and 150.3 μmol h^−1^ was observed for pristine MIS and 1T/2H MoS_2_ due to its quick recombination of charge carriers, as shown in Fig. 5a. Moreover, the loading of 1T/2H MoS_2_ to MIS resulted in the augmented hydrogen evolution rate of MMoS_2_-*x* composites owing to the synergistic effect of heterojunction created between both the individual materials and the increased metallic nature of 1T-MoS_2_ providing suitable availability of electrons with densely exposed active sites. The H_2_ generation rates for MMoS_2_-20, MMoS_2_-30, and MMoS_2_-40 were obtained to be 321.6, 370, and 298 μmol h^−1^, respectively. As per Fig. 5a, amongst all the synthesized composites, MMoS_2_-30 exhibited the highest H_2_ evolution rate, which is 2.7 times that of neat MIS. However, the decreased rate of H_2_ evolution by the overloading of 1T/2H-MoS_2_ ascribed that the active sites of MIS might get shielded by the black-coloured 1T/2H-MoS_2_ obstructing the absorption of photons. Fig. 5b presents the linearly enhanced photocatalytic H_2_ evolution of all the materials with increased time, with MMoS_2_-30 having optimum H_2_ evolution.

Furthermore, the photostability of MMoS_2_-30 towards photocatalytic H_2_ evolution was executed under the same reaction conditions for consecutive 4 reaction cycles, as displayed in Fig. 5c. The figure depicts no remarkable deviation in activity during the four reaction cycles, which verified the stability of the material for its practical applications. The apparent conversion efficiency (ACE) of MMoS_2_-30 was evaluated to be 5.9 by applying eqn (7),7
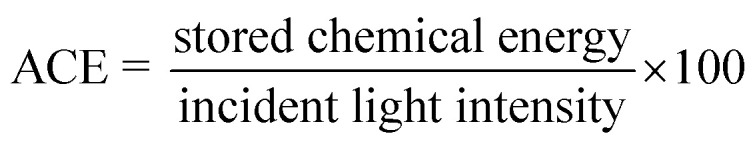


### Photocatalytic H_2_O_2_ evolution

**Fig. 6:**
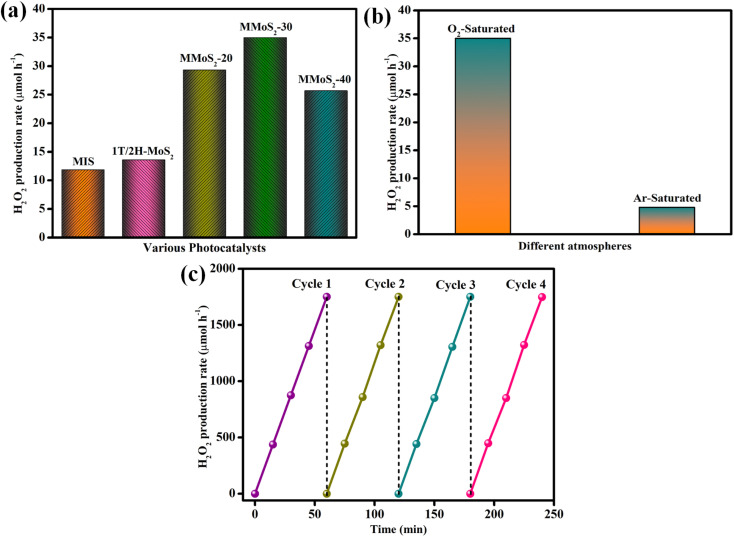
(a) Rate of photocatalytic H_2_O_2_ evolution by neat MIS, 1T/2H-MoS_2_ and MMoS_2_-30 photocatalyst. (b) Photocatalytic H_2_O_2_ production by MMoS_2_-30 composite under different atmospheres and (c) photostability test of the MMoS_2_-30 photocatalyst for photocatalytic H_2_O_2_ evolution.

The photocatalytic H_2_O_2_ production by the as-prepared photocatalysts was conducted in oxygen-bubbled water with visible light illumination for 2 h, and the results are presented in Fig. 6a. As shown in the figure, neat MIS and 1T/2H-MoS_2_ exhibited poor H_2_O_2_ generation rates of 11.84 and 13.6 μmol h^−1^ attributing to its quick recombination rate of e^−^/h^+^ pairs. The coupling of IT/2H-MoS_2_ nanoflower with MIS microflower remarkably enhanced the photocatalytic H_2_O_2_ production of MMoS_2_-*x* composites in the order MMoS_2_-40 < MMoS_2_-20 < MMoS_2_-30 after the formation of the Z-scheme heterojunction between both the neat moieties. MMoS_2_-*x* nanocomposites with increasing content of 1T/2H-MoS_2_, up to 30% with respect to the weight of neat MIS, exhibited increased H_2_O_2_ generation rates, *i.e.*, MMoS_2_-20 (29.34 μmol h^−1^) and MMoS_2_-30 (35 μmol h^−1^). However, with further addition of 1T/2H-MoS_2_ to 40%, a deviation in the photocatalytic H_2_O_2_ production efficiency was noticed at 25.7 μmol h^−1^ for MMoS_2_-40, indicating hindrance in the photon energy absorption due to the excess loading of 1T/2H-MoS_2_. The results demonstrated that lower and higher loadings of 1T/2H-MoS_2_ were subjected to decreased photocatalytic H_2_O_2_ production. Therefore, an appropriate amount of the pristine 1T/2H-MoS_2_ is essential for the improved charge carrier migration and separation, thus facilitating the photocatalytic performance of the MMoS_2_-30 heterostructure.

Since the photocatalytic H_2_O_2_ production reaction was executed under an oxygen-saturated atmosphere, a comparison study was performed under O_2_ and Ar-saturated conditions to examine the impact of oxygen on the H_2_O_2_ production rate. As displayed in Fig. 6b, the H_2_O_2_ generation rate of MMoS_2_-30 in the presence of Ar-saturated water is significantly lower than that in the oxygen atmosphere, suggesting that the oxygen atmosphere plays a pivotal role in accelerated H_2_O_2_ production.^54^ Furthermore, the stability of the synthesized material MMoS_2_-30 was determined by performing the reusability of photocatalytic H_2_O_2_ reaction for consecutive 4 cycles up to 480 min. Fig. 6c revealed that the prepared material maintains its stability as the photocatalytic activity shows no significant changes even after 4 successive reaction cycles.

After the assessment of the reusability test, XRD and XPS analyses were performed for the used MMoS_2_-30 sample, as illustrated in Fig. S5,† revealing negligible variation in the stability of the photocatalyst. The superior photocatalytic behaviour of MMoS_2_-30 nanocomposite in terms of H_2_ and H_2_O_2_ production has been contrasted with those of the alternative semiconductor-based photocatalysts under several parameters, and the outcomes are summarized in Tables S1 and S2,† respectively.

### Mechanistic approach

**Scheme 2:**
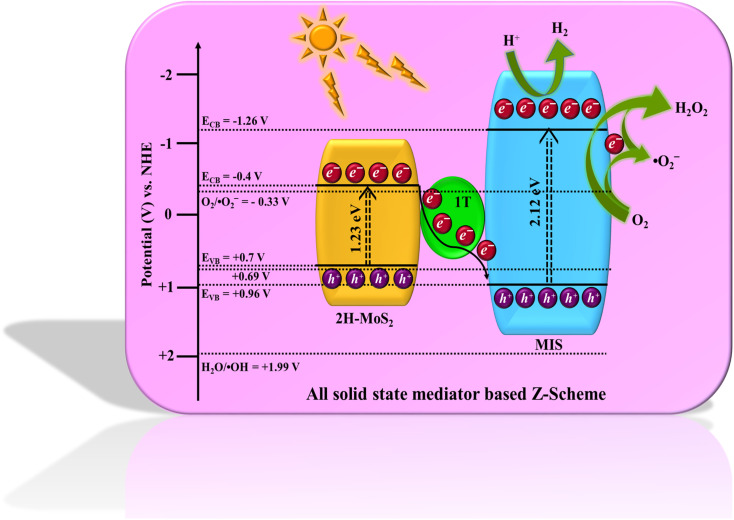
Schematic representation of the proposed mediator-based Z-scheme charge transfer mechanism of the MMoS_2_-30 photocatalyst for photocatalytic H_2_O_2_ and H_2_ production under visible light irradiation.

Based on the aforementioned experimental findings, a possible mechanism for the photocatalytic reaction (H_2_ evolution and H_2_O_2_ formation) of the as-prepared MMoS_2_-30 photocatalyst is envisioned and illustrated in Scheme 2. The combined result of UV-Vis DRS spectra and Mott–Schottky (MS) analysis reveal the band edge potentials for MIS, *i.e. E*_VB_ = 0.86 and *E*_CB_ = −1.26 V while for 1T/2H-MoS_2_, *E*_VB_ = 0.7 and *E*_CB_ = −0.4 V. Considering the band edge potentials, a bio-inspired solid state Z-scheme mechanism was followed by photo-induced electrons *via* the 1T-phase electron mediator. On exposure to visible light, both the individual materials are capable of developing photo-generated holes and electrons in their respective VB and CB. Propelled by the strong inherent electric field created in the MMoS_2_-30 photocatalyst, the photo-induced electrons move through metallic 1T-MoS_2_, transitioning from the CB of 2H-MoS_2_ to the VB of MIS by merging with the holes within the solid state Z-scheme heterostructure.^32,33^ Holes present in the VB of MIS oxidize ethanol to generate protons, increasing electron availability in the CB of MIS. The CB of MIS possesses a potential that is more negative than the redox potential of O_2_, enabling the production of H_2_O_2_ (−0.33 V for one electron two-step pathway or +0.69 V for two electron single step pathway).^24,45^ Consequently, the electrons present on the CB of MIS generate H_2_ from water reduction as the CB of MIS satisfied the potential required (H_2_O/H_2_ = −0.41 eV) for water reduction as displayed in Scheme 2.^55,56^ This process enhances the separation efficiency of exciton pairs, reducing their tendency of rapid recombination that leads to elevated photocatalytic activity. Furthermore, this Z-scheme mediated charge transfer pathway was substantiated by the shifting of binding energies of elements in MMoS_2_-30 from XPS analysis, as previously discussed. Additionally, scavenger test experiments were performed to clarify the mechanism mentioned above. To study the influence of ˙OH, h^+^, e^−^, and ˙O_2_^−^ in photocatalytic H_2_O_2_ generation, an array of scavenging agents comprising isopropanol (IPA), citric acid (CA), dimethyl sulfoxide (DMSO), and *para*-benzoquinone (PBQ) were added, respectively. According to Fig. S6a,† with the inclusion of DMSO and PBQ, a noticeable reduction in the efficacy of H_2_O_2_ generation occurred, indicating e^−^ and ˙O_2_^−^ to be the dominant active species towards O_2_ reduction *via* both the two-electron single step and one electron two-step pathway. Conversely, the participation of IPA and CA did not significantly impede the photocatalytic generation of H_2_O_2_. This implied that ˙OH and h^+^ are not the primary reactive species responsible for O_2_ reduction. Again, to investigate the Z-scheme charge transfer dynamics of MMoS_2_-30 towards the robust photocatalytic performance, NBT (nitroblue tetrazolium) test was carried out. As shown in Fig. S6b,† a decrease in the peak intensity of MMoS_2_-30 was observed compared to the pristine MIS, confirming the detection of a higher concentration of ˙O_2_^−^ radicals in the case of MMoS_2_-30. Further, electron paramagnetic resonance spin trap analysis (EPR) was conducted as a confirmatory test for ˙O_2_^−^ radical formation (Fig. S6c†). The quartet signal with a 1 : 1 : 1 : 1 ratio for MMoS_2_-30 was obtained in the presence of light, indicating the formation of ˙O_2_^−^ radicals. MMoS_2_-30 displayed no signal in the dark condition, attributing that in the presence of light, the availability of ˙O_2_^−^ radicals is higher over MMoS_2_-30 owing to the Z-scheme heterojunction.^57^ Hence, the synergistic effect developed between 1T/2H-MoS_2_ and MIS leads to the augmented photocatalytic activity for both H_2_ (370 μmol h^−1^) and H_2_O_2_ (35 μmol h^−1^) evolution process, which can be attributed to the following factors:

(i) Development of *in situ* generated 1T/2H-MoS_2_ nanoflowers over MIS microflowers offering a multitude of active sites for both H_2_O_2_ and H_2_ generation.^58^

(ii) The metallic 1T-phase in the heterostructure functions exceptionally as an electron reservoir and intermediary between 2H-MoS_2_ and MIS.^32^

(iii) Also, the intricate interactions between the neat counterparts, effective separation of exciton pairs with reduced recombination rate, enhanced current density, and lesser charge transfer resistance, as discussed in the PL, TRPL, transient photocurrent, and EIS measurements, boost the photocatalytic performance of the MMoS_2_-30 composite.

A detailed explanation of the successive steps followed by MMoS_2_-30 photocatalyst for photocatalytic H_2_ production (eqn (8)–(13)) and H_2_O_2_ generation (eqn (14)–(18)) are given below-equations for H_2_ production

8 ⇒ MMoS_2_-30 + *hν* → MMoS_2_-30 (e_CB_^−^ + h_VB_^+^)

9 ⇒ H_2_O → H^+^ + OH^−^

10 ⇒ MIS (e^−^) + 2H^+^ → H_2_

11 ⇒ CH_3_OH + h^+^ → ˙CH_2_OH + H^+^

12 ⇒ ˙CH_2_OH → CH_2_O + e^−^ + H^+^

13 ⇒ 2H^+^ + e^−^ → H_2_

Equations for H_2_O_2_ generation

14 ⇒ MMoS_2_-30 + *hν* → MMoS_2_-30 (e_CB_^−^ + h_VB_^+^)

15 ⇒ CH_3_CH_2_OH + 2h^+^ → CH_3_CHO + 2H^+^

Single-electron two step

16 ⇒ MMoS_2_-30 (e_CB_^−^) + O_2_ → ˙O_2_^−^

17 ⇒ MMoS_2_-30 (e_CB_^−^) + ˙O_2_^−^ + 2H^+^ → H_2_O_2_

Two-electron single step

18 ⇒ O_2_ + 2H^+^ + 2e^−^ → H_2_O_2_

## Conclusion

Concisely, MIS@1T/2H-MoS_2_ (*i.e.*, MMoS_2_-*x*) photocatalysts are successfully fabricated *via* a simple hydrothermal strategy without any surfactants where MIS microflowers are embellished with 1T/2H-MoS_2_ nanoflowers. In the proposed Z-scheme charge dynamics of the system, the 1T-phase serves as a high flux electron mediator. The designed hybrids were applied as efficient photocatalysts towards H_2_O_2_ generation and H_2_ production on exposure to visible light. Notably, MIS@1T/2H-MoS_2_ heterostructure with 30% loading of 1T/2H-MoS_2_ (*i.e.*, MMoS_2_-30) stands out with remarkable improvements in H_2_O_2_ yield reaching 35 μmol h^−1^ and H_2_ evolution rate of 370 μmol h^−1^ being 3 and 2.7 times higher than pristine MIS, respectively. The ameliorated photocatalytic activity characterized by reduced PL peak intensity, decreased arc radius in the Nyquist plot, extended TRPL lifespan, and elevated photocurrent densities can be attributed to the swift charge carrier separation facilitated by high electron transport *via* 1T-MoS_2_ in the Z-scheme charge kinetics. The incorporation of 1T/2H-MoS_2_ in small quantities amplifies the absorption capability of MMoS_2_-*x* photocatalysts, significantly contributing to their overall efficiency. Moreover, the promising photostability obtained from reusability tests of the MMoS_2_-30 hybrid validates its feasibility for practical utilization. This study offers a fresh outlook for the design and development of Z-scheme oriented noble metal-free co-catalyst-based heterostructure with appreciable potential for visible light photocatalysis.

## Conflicts of interest

There are no conflicts to declare.

## Supplementary Material

NA-006-D3NA00912B-s001
