# Supplementary material for: Augmented photocatalysis induced by 1T-MoS2 bridged 2D/2D MgIn2S4@1T/2H-MoS2 Z-scheme heterojunction: mechanistic insights into H2O2 and H2 evolution
Source: Nanoscale Adv. 2024 Jan 15;6(3):934–46. doi: 10.1039/d3na00912b (PMC10825931; doi:10.1039/d3na00912b)
Supplement: NA-006-D3NA00912B-s001 [file NA-006-D3NA00912B-s001.pdf]

Electronic Supplementary Material (ESI) for Nanoscale Advances.
This journal is © The Royal Society of Chemistry 2023

Supporting Information

Augmented Photocatalysis Induced by 1T-MoS_2 Bridged 2D/2D $MgIn_2S_4$@1T/2H-MoS_2 Z-Scheme Heterojunction: Mechanistic Insights for H_2O_2 and H_2 Evolution

Sarmistha Das, Lopamudra Acharya, Lijarani Biswal and Kulamani Parida*

Centre for Nano Science and Nano Technology, Institute of Technical Education and Research, Siksha 'O' Anusandhan University, Bhubaneswar-751030

**Corresponding author*

***Corresponding Author's Email:**

kulamaniparida@soa.ac.in

paridakulamani@yahoo.com

Tel. No.: +91-674-2351777 and Fax. +91-674-2350642

Characterization techniques:

Diffraction (XRD) analysis was carried out using a Rigaku Miniflex X-ray diffractometer equipped with a monochromator and Cu Kα radiation (λ = 0.154 nm, 30 KV/50 mA). For identifying the chemical states of the samples, X-ray photoelectron spectroscopy (XPS) was conducted with a VG Microtech Multilab ESCA-3000 spectrometer, employing Mg-Kα as the X-ray source. The morphological verification, including Field Emission Scanning Electron Microscopy (FESEM) was performed using a FEIN Quanta-400 FEG-SEM. The internal structure of the synthesized materials was analysed through High Resolution Transmission Electron Microscopy (HRTEM) using a JEOL-JEM-2100 transmission electron microscope. For determining the bandgap and absorbance characteristics, UV-Visible Diffuse Reflectance Spectroscopy (UV-Vis DRS) was conducted with a JASCO-V-750 UV-Vis spectrometer with $BaSO_4$ as the reference, covering a range of 200-800 nm. Photoluminiscence (PL) spectra including excitation and emission spectra were examined using a JASCO FP-8300 fluorescence spectrometer, employing an excitation wavelength of 325 nm and a xenon (Xe) lamp as the light source. The average life span of exciton pairs was examined through TRPL analysis using life spec II EDINBURGH PHOTONICS instrument.

Photoelectrochemical measurements:

A multi-channel Ivium potentiostat-galvanostat electrochemical workstation (IVIUM-N-STAT) was employed for photoelectrochemical measurements utilizing a three-electrode configuration consisting of a counter electrode (Pt foil), a reference electrode (Ag/AgCl) and a working electrode (fluorine-doped Tin oxide- FTO). 0.5 M H_2SO_4 and 0.5 M Na_2SO_4 aqueous solution was utilized as electrolytes for 1T/2H-MoS_2 and MIS. To prepare the working electrode, a mixture containing 10 mg of sample, 0.7 ml of ethanol and 1% nafion (20 µl) was coated onto the conducting surface of the FTO by drop-casting method. The FTO-coated electrode was then dried in an oven at 90 °C for 12 hours. The light source was provided by 300 W Xenon lamp equipped with a 400 nm cut-off filter.

Photocatalytic H_2O_2 production experiment:

The photocatalytic generation of H_2O_2 from pristine 1T/2H-MoS_2, MIS and MMoS_2-x photocatalysts was conducted under visible light exposure in an oxygen-saturated environment. Initially, 0.02 grams of the photocatalysts were dispersed in a mixed solution of 19 mL DI water and 1 mL ethanol through 10 min of ultrasonication to ensure complete dispersion.

Subsequently, the sample was exposed to O_2 gas for 30 min before being irradiated to 250 W visible light to create an oxygen equilibrated environment. After 2 hours of light exposure, the photocatalysts were collected from the solution by centrifugation and filtration. Then 1 mL of the resulting solution was mixed with 2 mL of KI (0.1 M) solution, followed by the addition of 0.05 mL of ammonium molybdate (0.01 M) solution to yield a light yellow colour. Then finally, the concentration of of H_2O_2 was determined by measuring the absorbance at a wavelength of 350 nm using an UV-Vis spectrophotometer.

Photocatalytic H_2 evolution experiment:

The photocatalytic water splitting reaction was performed inside a pyrex quartz glass photoreactor connected to a 150 W Xenon lamp emitting light with a wavelength greater than 420 nm. For the experiment, 20 mg of photocatalysts (1T/2H-MoS_2, MIS, $MMoS_2$-x composites) were dispersed in 20 mL of a 10% methanol solution (used as a sacrificial agent). The suspension was continuously stirred to prevent the catalysts from settling at the bottom of the reactor. Prior to exposing the system to light, the reactor's atmosphere was rendered inert by purging it with N_2 gas multiple times. After 1 hour of light exposure, the hydrogen gas produced was collected through water displacement method. The quantity of H_2 evolution was quantified using a GC-7890B (Agilent technology) equipped with a thermal conductivity detector (TCD) and a 5 Å molecular sieve packed column.

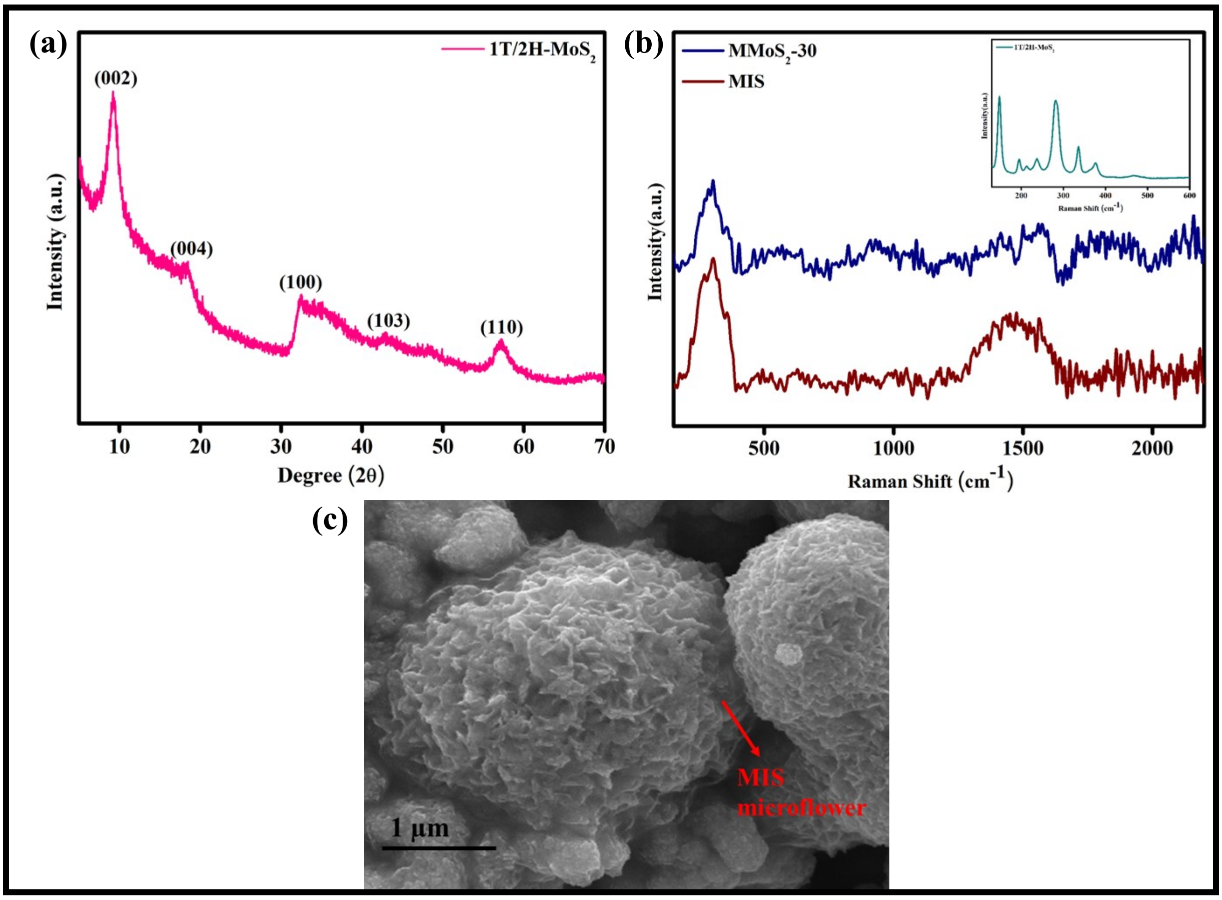

Figure S1. (a) XRD pattern of neat 1T/2H-MoS_2 (b) Raman spectra of neat MIS and $MMoS_2$-30 (Inset: Raman spectra of neat 1T/2H-MoS_2) and (c) FESEM image of MIS microflower.

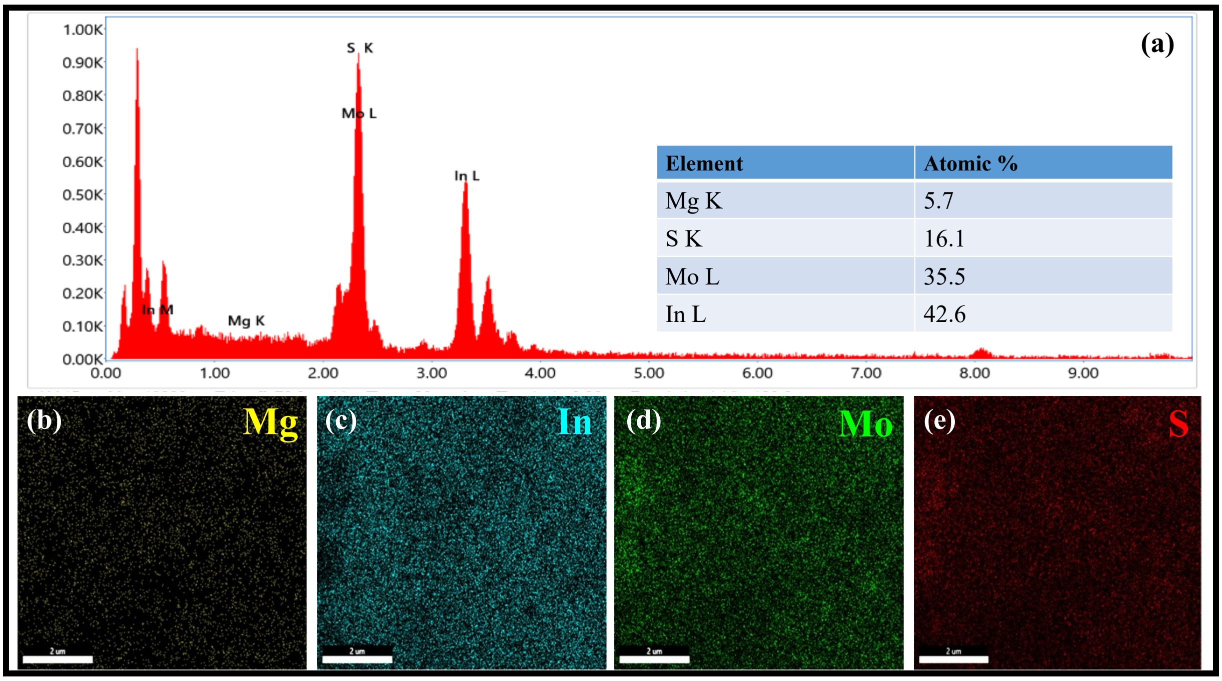

Element	Atomic %
Mg K	5.7
S K	16.1
Mo L	35.5
In L	42.6

Figure S2. (a) EDX spectra of $MMoS_2$-30 composite and (b) Elemental mapping of Mg, In, Mo and S elements for $MMoS_2$-30.

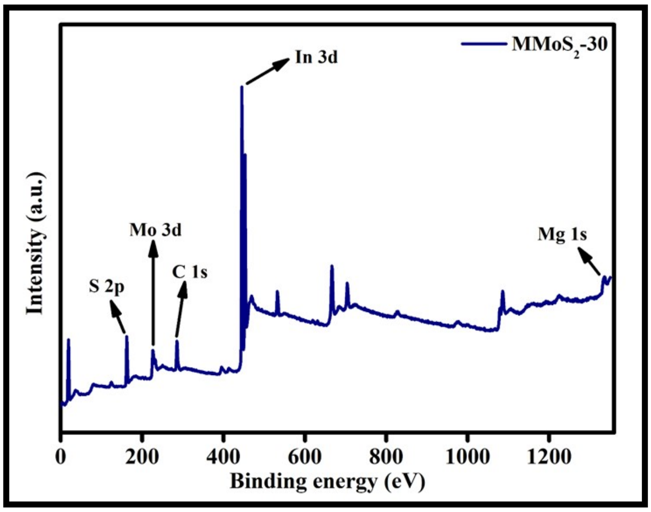

Figure S3. XPS survey spectra of $MMoS_2$-30 photocatalyst.

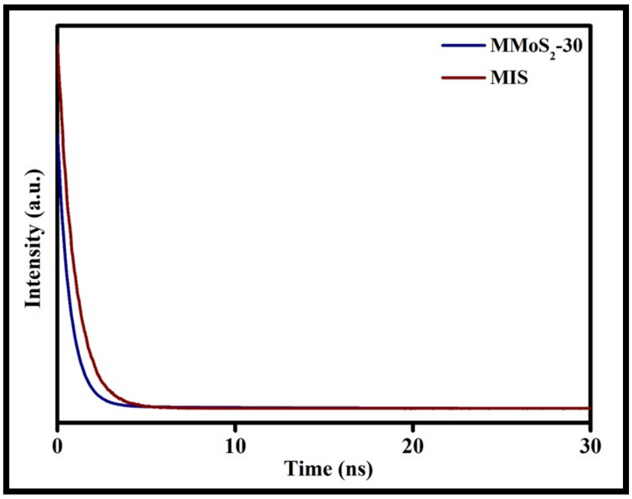

Figure S4. TRPL plot for neat MIS and $MMoS_2$-30 composite.

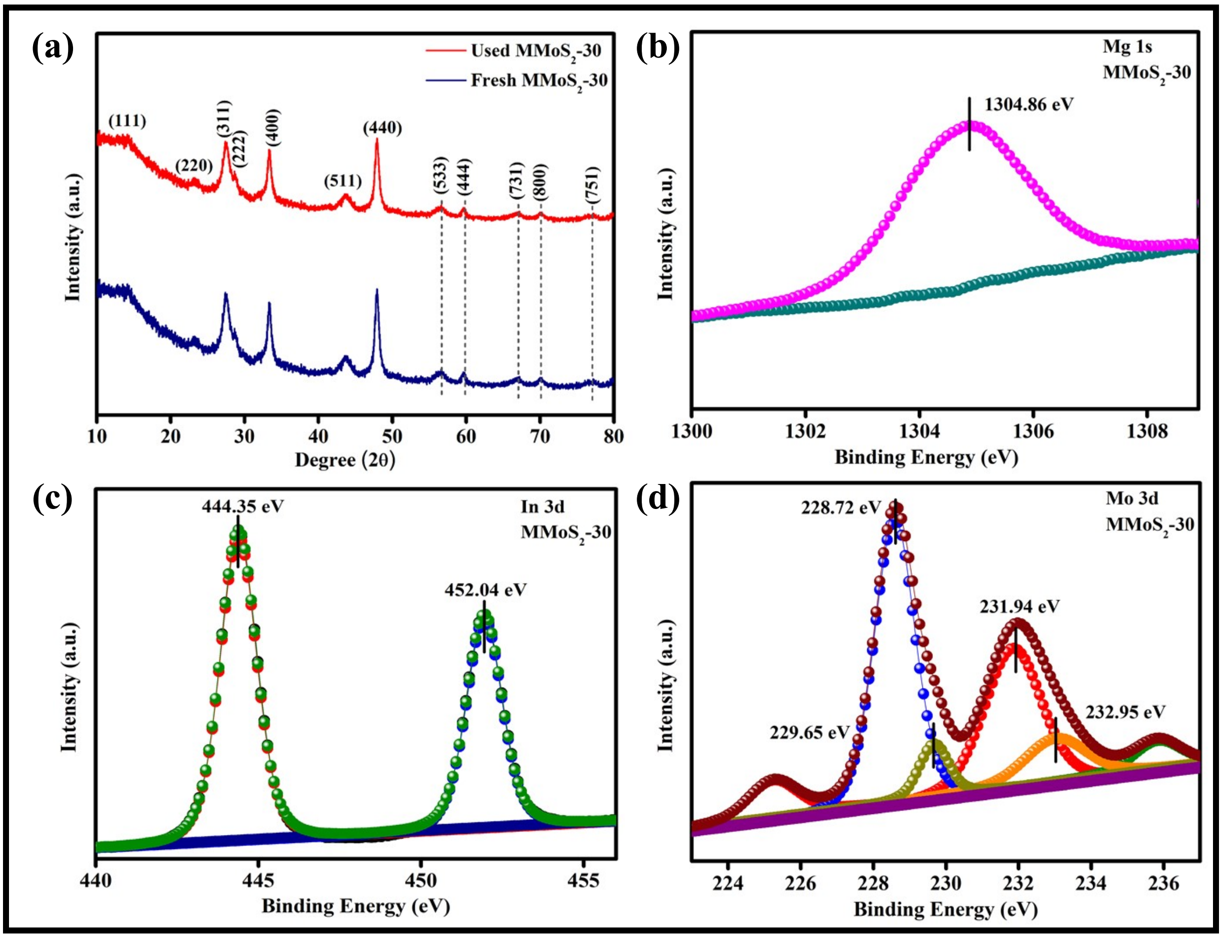

Figure S5. (a)XRD pattern for fresh and used $MMoS_2$-30 photocatalyst. After use XPS spectra for (b) Mg (c) In and (d) Mo in $MMoS_2$-30.

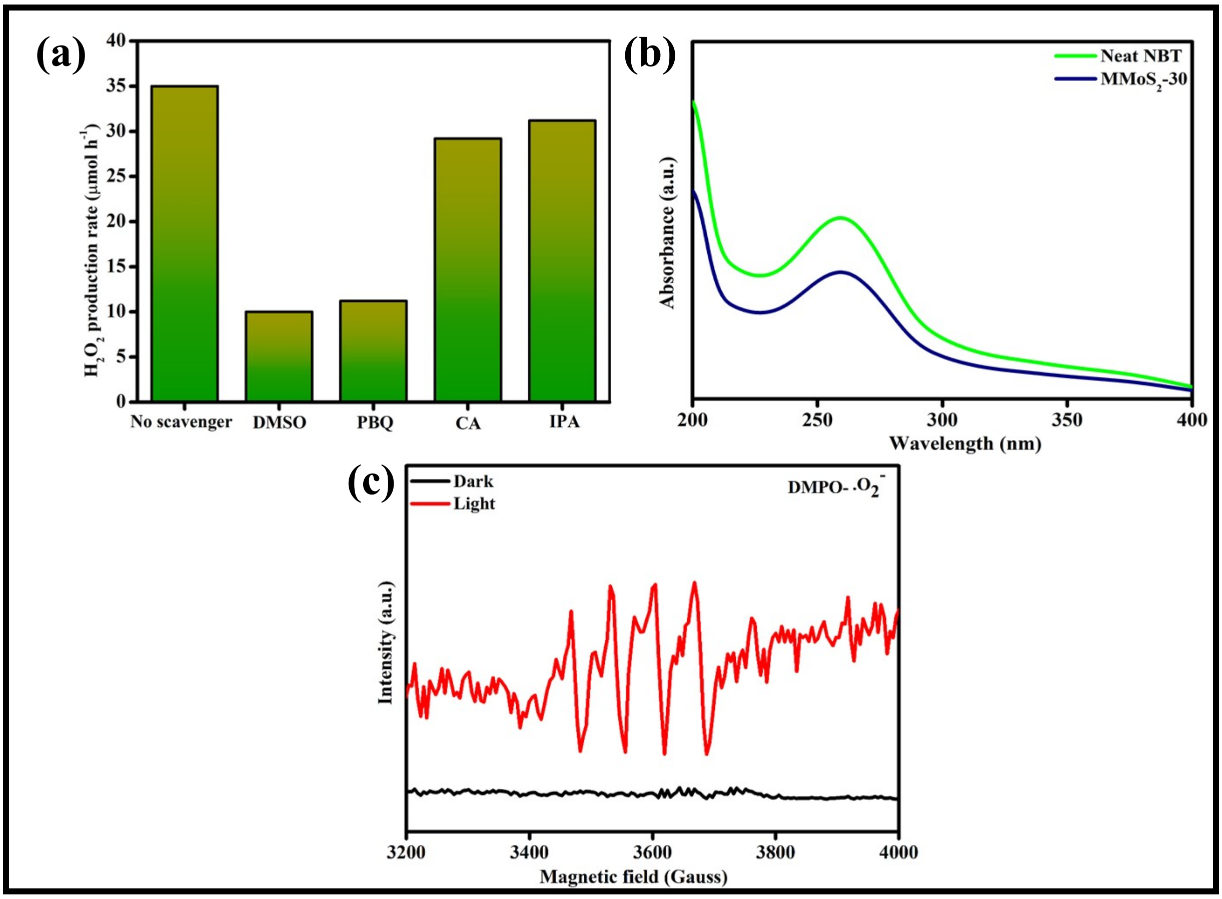

Figure S6. (a) Scavenger test for $MMoS_2$-30 towards photocatalytic H_2O_2 evolution. (b) NBT experiment for $\bullet O_2^-$ and (c) EPR spectra for $\bullet O_2^-$ in dark and light conditions.

Table S1. Comparative study of photocatalytic H_2 evolution

Sl.No.	Material	Amount of catalyst (mg)	Sacrificial agent	Light source	Rate of H_2 evolution	Reference
1.	1T/2H-MoS_2/O-g-C_3N_4	10	Triethanolamine (TEOA)	300 W Xe lamp	1841.72 $\mu molg^{-1}h^{-1}$	1
2.	1T/2H-MoS_2@CNT/CNU	40	OA solution	300 W Xe lamp	1563.32 $\mu molg^{-1}h^{-1}$	2
3.	1T-MoS_2/Au/S-doped g-C_3N_4	15	Methanol	300 W Xe lamp	4708.3 $\mu molg^{-1}h^{-1}$	3
4.	CN/MoS_{2-x}	25	Triethanolamine (TEOA)	300 W Xe lamp	441.3 $\mu molg^{-1}h^{-1}$	4
5.	MoS_2@Ag_2S	10	0.1M Na2S, 3M NaCl, 0.04M	300 W Xe lamp	3516 $\mu molg^{-1}h^{-1}$	5

			Na2SO3			
6.	NH_2-MIL-125(Ti)/O,P-MoS_2	15	Acetonitrile, TEOA, DI	LED light	339.3 μmolg^{-1}h^{-1}	6
7.	MIS/e-BCN	20	Methanol	Visible light	330 μmolg^{-1}h^{-1}	7
8.	$MMoS_2$-30	20	0.35M Na2S and 0.25M Na2SO3	Visible light	370 μmolg^{-1}h^{-1}	Current work

Table S2. Comparative study of photocatalytic H_2O_2 production

Sl.No.	Photocatalysts	Light source	Time duration	Rate of H_2O_2 production	Reference
1.	MoS_{2-V}/TCN	300 W Xe lamp	60 min	1879 μmolg^{-1}h^{-1}	8
2.	MoS_2/FeS_2	300 W Xe lamp	60 min	1.5 mmol g^{-1}h^{-1}	9
3.	1T-MoS_2/g–C_3N_4	Simulated solar (AM 1.5)	60 min	42 μM	10
4.	Fe_2O_3@C@1T/2H-MoS_2	Visible light	-	1575 μmolg^{-1}h^{-1}	11
5.	$MMoS_2$-30	Visible light	120 min	35 μmolh^{-1}	Current work

References

1. H. Xu, J. Yi, X. She, Q. Liu, L. Song, S. Chen, Y. Yang, Y. Song, R. Vajtai, J. Lou and H. Li, *Appl. Catal. B: Environ.,* 2018, **220**, 379-385.
2. C. Sun, F. Li, J. Ren, J. Wu, G. Wang and L. Chen, *Appl. Surf. Sci.,* 2021, **569**, 151072.
3. D. Li, G. Zeng, Y. Wu, Z. Zhou, W. Guo and C. Li, *Int. J. Hydrog. Energy.*, 2023.
4. Y. Xu, J. Ouyang, L. Zhang, H. Long, Y. Song and Y. Cui, *Chem. Phys. Lett.*, 2023, **814**, 140331.
5. C. J. Chang, Z. T. Tsai, K. S. Lin and Y. H. Nian, *J. Photochem. Photobiol. A.,* 2023, **445**, 115027.

6. L. Wang, Y. Hu, J. Xu, Z. Huang, H. Lao, X. Xu, J. Xu, H. Tang, R. Yuan, Z. Wang and Q. Liu, *Int. J. Hydrog. Energy*., 2023, **48**, 16987-16999.
7. L. Acharya, G. Swain, B. P. Mishra, R. Acharya and K. Parida, *ACS Appl. Energy Mater.*, 2022, **5**, 2838-2852.
8. G. Jiang, X. You, B. An, B. Zhu, F. Liu, X. Duan, Y. Wang and R. Zhao, *Appl. Surf. Sci*., 2023, **618**, 156656.
9. Y. Yang, H. Yu, M. Wu, T. Zhao, Y. Guan, D. Yang, Y. Zhu, Y. Zhang, S. Ma, J. Wu and L. Liu, *Appl. Catal. B: Environ.,* 2023, **325**, 122307.
10. X. Hu, X. Zeng, Y. Liu, J. Lu, S. Yuan, Y. Yin, J. Hu, D. T. McCarthy, and X. Zhang, *Appl. Catal. B: Environ*., 2020, **268**, 118466.
11. Y. Yang, Q. Wang, X. Zhang, X. Deng, Y. Guan, M. Wu, L. Liu, J. Wu, T. Yao and Y. Yin, *J. Mater. Chem. A*., 2023, **11**, 1991-2001.
